# Book Reviews

**Published:** 1813-11

**Authors:** 


### CRITICAL ANALYSIS

### *OF RECENT PUBLICATIONS*

IN THE

### DIFFERENT BRANCHES OF PHYSIC, SURGERY, AND MEDICAL PHILOSOPHY.

*Appendix to Observations on the Contracted Intestinum Rectum: containing some additional Facts relative to that Complaint; with several Cases, and two Engravings.* By W. White, Member of the Royal College of Surgeons, London, and one of the Surgeons to the City Infirmary and Dispensary, Bath. 8vo. Bath, 1813. pp. 53.

SOME time ago we gave an analysis of Mr. White's treatise on Contracted Intestinum Rectum. The ingenuity and practical utility of that treatise, we endeavored to impress on our readers. The subject, always of importance, had been brought more under public observation than heretofore, by the publication of Mr. Copeland; the immediate inducement, as it then seemed, to Mr. White's pursuing the inquiry. Since that period many cases have fallen under the care of Mr. White, nine of which are here related.

There are two points in this little volume principally to be looked to: one of these is a practical fact of some importance; the other assumes more the character of an hypothesis, though it is not directly hypothetical.

A marked trait in the contracted rectum, and constituting a prominent feature in the diagnosis, is that of the fæces being found to be lessened in their diameter. The occasional deviation from this, in advanced stages of the disease, when fæces of a natural size, at times, pass, Mr. White notices, with a view to prevent the practitioner mistaking the cases when this circumstance does arise; and as it appears mechanically contradictory, he enters into the following explanation of it.

"If the stricture should happen to be low in the rectum as not to allow room for the accumulation of fæces, it must appear evident that they they will be found uniformly small in diameter, in proportion to the degree of stricture, while they continue to be discharged in a figured state. And also, when the stricture happens to be high up the rectum, so long as the gut below the stricture retains its natural expulsive power, an accumulation will be prevented, and the diminished size of the fæces will continue. But as the disorder increases, the inferior portion of the intestine gradually loses that power; so that when the contraction becomes considerable, only a small quantity of fæces pass at a time through the stricture, and not being sufficient to stimulate the lower part of the rectum, an accumulation goes on from time to time, until at length it becomes difficult to remove; when on these occasions fæces of a natural size have sometimes been discharged."

It has now some time been the fashion, or rather, perhaps, has now become an admitted fact, that strictures in the urethra, by some, yet unexplained, sympathy, produce morbid actions in distant parts of the system, of a character, *à priori*, not at all referable to such a cause. Mr. White does not doubt but strictures of the rectum may excite derangement in remote parts of the intestinal canal, or in organs that are associated with it in the performance of its natural functions. The *liver* is the organ to which our author looks as most likely to undergo this sympathetic derangement. In two patients a tuberculated state of the liver was believed to be the consequence of stricture in the intestine; and as the opinion is in a measure new, we shall lay before our readers the whole of the third case, which goes, in the author's opinion, particularly to this point. In this the biliary secretion was disturbed, and the alvine evacuations were always of a light clay color, except when the patient was taking calomel.

"Case 3.—Feb. 1812, E. Morgan, an unmarried woman, sixty-three years of age, complained of having been subject to pains about the os sacrum shooting down the hips, between four and five years. She had been always of a costive habit of body, seldom having any evacuation for four or five days, and not then without the aid of a strong purgative medicine. About a year ago she was attacked with a sudden hæmorrhage, which she supposed to have been a return of the catamenia in a most extraordinary and violent manner; but on the hæmorrhage recurring shortly after, she was convinced the discharge proceeded from the rectum; ever since which she has had frequent returns of the hæmorrhage; and upon that ceasing, a serous discharge supervened. Between five and six months ago she began to experience considerable pain and difficulty in passing her stools, attended with tenesmus, and almost constant pain in the gut: her strength was much reduced, with frequent flushings of heat, but her pulse was regular.

"On examination, I found great irregularity and induration in the rectum, about an inch from the anus, which extended some way up the the gut, when a considerable contraction was discovered, but yet a sufficient passage to admit the tip of the finger being introduced: the contracted part had an irregular and indurated feel. That I might have the patient more immediately under my care, she was admitted an in-patient at the Bath City Infirmary on the 25th of February. The next day a small tent was introduced, and the following pills were prescribed:

℞. Extr. conii, ʒiſs.

Pil. hydrarg. ʒſs. M. f. pil. xxx æquales divid.

quarum capt. ij. mane et vespere.

" A clyster, with gruel and castor-oil, was also directed to be thrown up daily. Her diet—gruel, broth, arrow-root, and light puddings.

" Feb. 27th.—Has had several motions without the injection, and less pain—tent again introduced.

Capt. pil. extr. conii, et pil. hydrarg. j. mane et vespere, et quoque pil. opiat. gr. j. o. n. h. s.

" 28th.—Has a troublesome cough, breathing short, with wheezing. Omittr. pil. hydrarg. &c.

℞. Liquor ammon. acet.

Aq. menth. pip. āā ℥iſs.

..... puræ, ............ ℥iij.

Syr. papav. alb, oxym. scillæ, āā ʒij. M. f. mist. Capt. coch, ij. ampl. 4ta. quaque hora. Repr. pil. opiat.—A tent introduced.

" 29th.—Breathing rather better, and less pain in the rectum. Repr. mixt. et pil. opiat.—A tent introduced. As the bowels had not been freely open, an injection was directed.

" March 1st.—Had a good night; the bowels have been moved in consequence of the injection, with scarcely any appearance of blood—A tent introduced.

" 2d.—Breathing much worse, and cough more troublesome—pulse quick: has had two or three small loose motions without any blood. Repr. med. et enema laxativ.

" 3d.—Her breathing better, and cough not so troublesome: had three motions from the injection, but no blood.

" 4th.—Much the same: has frequent loose stools, (so as to prevent introducing the tent) but unattended with pain.

" 5th.—She has still a frequent discharge of loose stools. Injecr. enema opiat.

" 6th.—Breathing much worse, with increase of wheezing, and the cough more troublesome; skin hot and pulse quicker; tongue white, and complains of thirst. A very large quantity of consistent fæces has passed. Applicr. emp. canth. sterno. Repr. mixtur.; add spt. æther vitriol comp. ʒij.

" 7th.—Breathing somewhat relieved, but the feverish symptoms continue. Has had two small loose motions. Repr. mist. et capt. haust. anodyn. h. s.

" 8th.—Both breathing and cough better; pulse not so quick, and tongue cleaner. Has had three small motions with a little blood. Repr. enema opiat. On introducing a tent, I perceived a fœtid discharge from the rectum, which I had not before noticed. Repr. med.

" 11th.—

"11th.—Has very little uneasiness in the rectum, but general pains over the abdomen. Cough and breathing still troublesome, though in a slighter degree. Not so much heat on the skin, nor quickness of pulse. Repr. med. et enema opiat.

"12th.—Less pain over the abdomen. Although there is less heat on the skin, she complains more of thirst. Has had some small loose motions. Repr. enema laxativ.

"13th.—Has very little pain in the abdomen. The clyster occasioned several loose motions, which very much relieved her—a tent introduced.

"14th.—Breathing more affected; has had several loose motions. Applicr. emp. canth. sterno, et repr. med.

"15th.—Breathing somewhat relieved, but the cough still troublesome: has had two loose motions, besides what is found to pass away involuntarily on returns of cough—a tent introduced.

"16th.—Had a restless night, from the difficulty of breathing and cough: passed several sanious colored loose motions. Repr. med.

"17th.—Breathing and cough much the same, but now attended with an expectoration free and copious—has had two small loose motions of the same appearance as last. Repr. med. et enema laxativ.

"18th.—Breathing much the same; a little bloody mucus is brought up with the cough: has had more uneasiness in the bowels. Two injections have been given without producing any effect—the injection was ordered to be repeated with the addition of a little murias sodæ.

"19th.—Had no evacuation until she took some castor-oil this morning, which procured several motions: cough and breathing much the same. Repr. med.

"20th.—Had a better night: breathing not so difficult; skin cool; pulse regular; tongue clean. Passed three stools without any pain. Repr. med.

"22d.—Her breathing much better, and cough not so urgent: had a very good night; bowels open—a small tent introduced.

"24th.—Continues better: bowels still in an open state, and the evacuations of a more natural consistence—a tent introduced.

"26th.—Bowels having been confined yesterday has taken castor-oil, which procured three motions, one of them very copious—a tent introduced.

"27th.—Her breathing and cough better: bowels open—felt a soreness in the rectum after the tent yesterday.

"28th.—Feels better: has had two motions without pain—a tent introduced.

"30th.—Having had no evacuation yesterday took castor-oil, which operated two or three times.

"31st.—Complains of sickness, and the having brought up bile; had a motion this morning, followed by a little blood. The fœtid discharge from the rectum has ceased.

Capt. mist. salin. card. ℥i. 4tis. horis. Capt. haust. Anodyn. h. s.—a tent introduced.

"April

" April 3d.—Sickness better: complains of pain over the abdomen. Took castor-oil yesterday, which procured several motions—a tent introduced.

" 5th.—Less pain in the abdomen: bowels open, and the fæces discharged without pain—a tent introduced.

" 9th.—A tent introduced.

" 11th.—Complains of having had a considerable soreness in the rectum since the last tent was introduced. Although, on examination with the finger, the contraction does not appear increased, yet there is a greater difficulty in passing the tent from the extreme irregularity on the internal surface of the gut—the tent was omitted. The bowels were kept open with castor-oil, and the evacuations continued to be discharged without pain or appearance of any blood. Her general health appeared also to be improving, and she was able to sit up a few hours daily, which she had not been able to do for a long time: her appetite was so much better as to render her very desirous of having a little animal food, which was complied with.

" On the 23d a tent was introduced, but could not pass it until I had previously ascertained the direction of the contracted part by introducing the finger, the irregularity of the surface continuing the same.

" 25th.—The tent occasioned considerable pain in the rectum, and a little blood followed its removal. She took castor-oil this morning, not having had a motion since the last tent was introduced.

" 26th.—Had several motions yesterday, and her bowels very open to-day: does not complain of any particular pain.

" 28th.—A small tent again introduced—the last time.

" 30th.—Complains of having had much soreness in the rectum since the last tent was introduced, and has had no motion. Rep. enema laxativ.

" May 1st.—Passed several motions. Had appeared to be rather weaker, her appetite having failed for the last day or two; but no material alteration was observable until the 5th, when, on entering the ward in the morning, I was surprised to find so great a change in her countenance; her breathing short; pulse extremely feeble; with every other appearance of a speedy dissolution. She died the same afternoon. The nurse informed me she had become suddenly worse in the night.

" *Appearances on dissection.*—On dividing the parietes of the abdomen, there were evident marks of peritoneal inflammation, and the intestines also exhibited a similar appearance, but more particularly the ilium, and its folds were glued together in several places, the consequence of inflammatory exudation; and on its surface there were different patches of coagulable lymph; there was also some purulent matter in the pelvis. On separating the rectum from the sacrum, its posterior part gave way, appearing that only the peritoneal coat at this part of the intestine had remained; the other coats having been destroyed by ulceration. The internal surface of the gut was extremely irregular, and its inner membrane entirely destroyed by ulceration; which process had extended somewhat less than an inch from the anus, as far as the contracted portion of the rectum. The

 muscular muscular coat was very much thickened and indurated, exhibiting the usual cancerous appearance: and in other places (besides the posterior part already noticed) it appeared to be entirely destroyed, as well as the inner coat, by the ulcerative process. At the termination of the ulceration there was a considerable contraction of the gut, from the diseased state of the muscular coat having formed a complete thick cartilaginous ring; and a little below it the jagged edges of the inner coat projected; its lower portion, as before mentioned, being entirely destroyed by ulceration. Above the cartilaginous ring the intestine was somewhat dilated, its inner membrane having an inflamed appearance, which had extended about two inches up the gut. The muscular and peritoneal coats, at the back part of the superior portion of the rectum, were thickened and indurated, extending in a line along the sacrum for nearly three inches above the contraction; the thickening gradually lessening as it extended upwards. A great quantity of solid fæces was collected above the contracted part, and properly tinged with bile.

" About the middle of the convex surface of the liver there was a very large tubercle, with several lesser ones dispersed throughout its substance.

" The fundus uteri was red, and the fimbriated extremities of the fallopian tubes were in a state of ulceration; no doubt from having been exposed to the purulent matter which was collected in the pelvis.

" The lungs had a diseased appearance, and with some difficulty separated from the back part of the thorax."

We learn from the introductory part, that the alvine discharges were always of a light clay color in this woman, except when calomel was taken; but in the detail of the case, it does not appear that calomel was given under the direction of Mr. White, or the fæces noticed as regards color. Dissection certainly showed a tuberculated state of liver; but neither the history or dissection remove the hypothetical character of the opinion. The investigation will probably be further pursued, and the sympathetic connection between strictured rectum and tuberculated liver, we hope, will be established on more extended evidence.

---

*Observations on Peritonitis, and on some other internal Inflammatory Affections.* By THOMAS SUTTON, M.D. of the Royal College of Physicians, late Physician to the Forces, and consulting Physician to the Kent Dispensary.

(*Continued from p. 336* )

THE merit of the author in this part of his work, which consists in a relation of cases, is principally founded on his directing a successful mode of practice in complaints which often prove tedious, difficult of cure, and even fatal. A case or two will show the value of the author's observations and practice.

"A gentleman consulted me on the subject of some obscure pains he experienced on the right side, midway between the ribs and fore part of the ilium, which were not then increased upon pressure. He said he had, for some time, labored under a disease which his medical advisers in town had not formed any precise opinion about; but he had been cupped, used the warm bath, and had taken opening medicines, without perceiving any other than temporary advantage. He had since applied to another source, and been dissatisfied with the result. The patient complained that he felt uneasiness with any thing of a shaking motion; but as he came to me in his carriage, and did not immediately meet with me at home, he had driven about under the idea of falling in with me. I found his countenance good, the pulse natural; but he expressed himself to be extremely uncomfortable and low, with depression of spirits, want of appetite, and when he did eat he became worse after it. I directed some opening medicines, and a soap plaster to be applied to the part affected; but, before the medicines could be got home, I was sent for, and found the patient in considerable pain, augmented on pressure. He was directed leeches and fomentations to the part, and afterwards a blister, and the bowels to be freely acted upon. The pain wore off to a certain extent; but in two days I was sent for in some haste, and found the patient in great pain, in a profuse clammy sweat, with a pulse of 120. Under these circumstances, I judged it proper to recommend blood to be drawn to sixteen ounces, which quantity was exceeded, and which afforded much relief, though the blood was not sizy, but tough. From this time the disease gradually got better, but returned twice, with the interval of a fortnight between the attacks. The third being violent, I considered it to be adviseable again to recommend venesection to some amount, which occasioned relief; but the second attack was comparatively mild, and the loss of blood was trusted to leeches. The other part of the treatment consisted in the use of warm fomentations, blisters, and purgative and aperient medicines, chiefly with sulphate of magnesia, and infusion of senna. The patient has had no further severe returns of this disease, though for some time he was frequently unwell, and obliged to use much caution in his mode of living and exercises. The disease, as appears, came on in a lurking obscure manner, was some considerable time before it arrived at a violent paroxysm, and was not subdued for upwards of six weeks, though much abated in the intervals of the attacks.

"The expressed seat of the disease was at a considerable distance from the kidnies, and in a line drawn from the anterior spinous process of the ilium, parallel to the linea alba. The seat of the disease is more particularly stated, because, previous to these violent attacks, the patient had perceived small quantities of blood to be discharged once or twice with the urine, though nothing of the kind had occurred during the period just detailed, when the secretion was copious, of proper color, and varying as it is known to do in attacks of fever, and was every day inspected while any urgent symptoms remained. Afterwards the appearance of blood in the urine was again occasionally observed, though in inconsiderable quantities. This discharge was probably excited by the same cause as a similar one which was noticed in the following case, though originating from different organs."

"Mr. Hurt, apothecary and chemist, residing on Deptford Bridge, about a month previous to the attack, which will be more particularly adverted to, consulted me on the subject of a disease which had been very painful to him for some time, and of which this was the second attack, after a short interval. He described it to be about an inch and an half round the navel. He had been advised to apply leeches, to use fomentations, and to blister the part; from which remedies he thought himself somewhat relieved. Under the circumstances I found him in, I recommended him to keep his bowels well open, and if there should be any material return of the disease, to have blood drawn from the arm to the extent of from sixteen to twenty ounces, which the present state of the habit appeared to me to be capable of allowing. In about a month from this time I was again desired to see him. He stated, that he had never been well from the complaint he consulted me about, but that now he was in great torture. He had considerable febrile heat, coated tongue, and a pulse one hundred and ten and upwards, with nausea and vomiting. The local affection was in extent nearly circular, with a diameter of about three inches, making the navel its centre. The sensation of the patient was that of a burning heat, with shooting pains, and a feeling of great weight in the part. The patient had had recourse to leeches, blisters, and fomentations, as before, but with no effect. I recommended blood to be drawn to the extent of sixteen ounces, and the bowels to be kept open. He was afterwards bled five times, to twelve, ten, and eight ounces, as the symptoms appeared to require, without any satisfactory progress being made. In addition, the part was fomented with the decoction of poppies. This was the treatment pursued during the first week of my attendance, and to about the tenth day of the violent return of the disease. It was next determined to try the effects of more active purgatives than had hitherto been done, with the addition of small doses of opium; the latter for the purpose of relieving pain, and repressing the tendency to vomit. By this plan, the bowels appeared to be completely evacuated of their contents, but without any material amendment ensuing. Recourse was then had to considerable doses of opium to relieve the disease, which had certainly the effect of mitigating pain, while the patient was under its powerful influence; but it did not appear to diminish the disease otherwise. Local bleeding was then employed again, and a blister applied, but these produced no perceptible advantage. The disease had now continued about three weeks. The local affection was very little abated; the pulse was somewhat diminished in frequency; and a fulness and hardness painful to the touch in the part affected, within the limits above described, had become much more evident, and particularly attracted the attention of the patient. Under these circum-

 stances, stances, I suggested the use of a common emollient poultice, but found it increased the local affection considerably. On this report, I determined to recommend the patient to employ a lotion composed of equal parts of aq. ammoniæ acet. and water, with half an ounce of rectified spirits to eight ounces. In two days afterwards, I called, and was gratified to find the patient much amended. He stated, that, from the very first application of the lotion, he had experienced relief, and that his pain returned only occasionally, wheu the use of the lotion repressed it. From this time the patient might be considered to be convalescent. The only things afterwards advised, were to keep the bowels open, to be moderate in the use of food, to avoid fermented liquors, and to use the lotion as frequently as might be found necessary. Since which time, (about a year,) I have uniformly heard a good account of Mr. Hurt's health."

Several other cases are related, attesting the good effects of cold applications in peritonitis, and in some affections of the chest. One case even proves the advantage of cold sea-bathing in consumption. We were much gratified with this part of the work, and congratulate the author on his resolution in combating both vulgar and professional prejudice, as well as upon the success which attended his practice. What will the hot-house practitioners think of applying a cold lotion to the chest in consumptive cases, which Dr. Sutton very coolly proposes? He has favored us with some interesting particulars of the practice of Dr. Stewart, a clergyman, who has obtained such high reputation in Scotland for curing consumption, as to attract the notice of a noble family in London, who consulted him in consequence of his decided success. Will it be credited that part of his plan consists in applying cold vinegar and water to the chest daily, which he also recommends to be well rubbed?

The injurious consequences of wearing too much flannel are illustrated by a case in which the patient consulted Dr. Sutton on account of a cough which had troubled him for some time, and by which he was becoming weak and emaciated.

"After an attendance of about ten days, the symptoms had grown worse. The patient was now so weak that he could with difficulty leave his bed: he had a very harassing cough, with an expectoration of an uncertain character, hectic fever, and profuse perspirations, with a pulse of one hundred and twenty. About this time, in one of my visits, the conversation turned on the state of the patient's health some years before, which was represented to be delicate, and that he was subject to rheumatic affections in various parts of his body, on exposure to cold. On this account, he wore great quantities of flannel, which led me to inquire into the state of his present clothing. I found he was completely invested in flannels of various and the warmest kinds, and that he constantly wore several such garments.

 As

As it appeared to me that this sort of clothing must be very prejudicial to him in his present state, and particularly by encouraging profuse perspirations, I recommended all but one of these garments to be dismissed, and, as soon as possible, to substitute for the remaining one a coarse calico waistcoat. After this change had been adopted, the patient very rapidly and completely recovered, which was plainly due to the diminished heat on the surface."

The remaining portion of the volume is devoted to the consideration of gout; and if personal experience is a recommendation, the doctor's authority is good, for his own sufferings in that complaint induced him to pursue bold and active treatment. In fact his own cure forms the chief part of the essay. He commences with some general observations upon the disease, which tend to dispose the reader to admit that something more may be done both in alleviating the pain, and even curing the disease, than merely leaving the patient to nature and despair. The remarks on this subject are supported by various authorities, and we fully concur with him in opinion that much may be done towards mitigating the violence, and removing the complaint, at least for a time.

The remedies from which he experienced the most decided benefit were strong purgatives. He afterwards, however, was so situated that he could not conveniently take them, and had recourse to a large dose of laudanum, which completely succeeded in subduing his gouty paroxysm.

From some experience, and also having seen the practice adopted by others, we have long been of opinion that opium taken in doses sufficiently large to ease the pain and induce sleep, followed up by suitable and adequate purgatives, are strongly to be relied upon in all cases of gout where the practitioner sees no indication to forbid their use. We are, therefore, pleased to find this practice, which at the same time we must remark is no new practice, corroborated by the statement of Dr. Sutton.

---

*A Practical Synopsis of Cutaneous Diseases according to the Arrangement of Dr. Willan, exhibiting a concise View of the Diagnostic Symptoms, and the Method of Treatment.* By THOMAS BATEMAN, M.D. F.L.S. Physician to the Public Dispensary and to the Fever Institution.—8vo. pp. 342. Longman and Co., 1813.

CONSIDERING the advances made by the ancients towards a knowledge of cutaneous affections, it seems remarkable that so little should have been accomplished by the moderns in this interesting department of medical science. To the ancients cients we are indebted for most of the names which at present distinguish cutaneous diseases, and to such authority, till very lately, we were compelled to resort for their description and treatment. Dr. Willan, the latter end of last century, presented us with the first number of a voluminous work upon this subject, of which he did not live to complete more than half the intended series. But that half was the most important; the general outline was sketched, though the master-hand was only put to the first part. In this publication he displayed an acquaintance with the writings of the ancients highly creditable to his erudition, and evinced an acuteness of perception, an accuracy of distinguishing, combined with scientific arrangement, worthy of the enlightened physician. From the rude mass of heterogeneous matter before him, he organized a rational system, and enriched it with new observations of his own. The difficulty of the subject is demonstrated by the fact, that eager as well-informed medical men usually are in the pursuit of objects which may signalize them, the subject of cutaneous diseases had received very little elucidation since the obscure writings of the Arabians, until Dr. Willan happily selected it as deserving his attention, and his success has shewn that his talents were equal to the laborious investigation. It is true that most practitioners were in some degree acquainted with the appearances, and knew something of the mode of treating some of the more frequent and prominent cutaneous diseases; but the most candid will be ready to allow that they often guessed at what is now reduced to certainty, and often acted empirically when they might have proceeded upon indubitable and scientific principles. If we were for a moment to admit, which indeed we cannot in truth, that the practice is not materially improved, there can be no doubt whatever that the knowledge of the nature and progress of cutaneous complaints is much advanced by Dr. Willan's valuable work. We can now at least promptly decide what the event of many cases will be, whether they are curable or not; and it is of not the least importance too, to give them a name, about which, what with the plates, the definitions, and the descriptions, we need not long hesitate. But Dr. Willan having projected eight orders of cutaneous diseases, only finished the description of four; and here would have been a lamentable deficiency indeed, had not an able successor in this line of practice immediately presented himself to occupy the chasm which would otherwise have occurred. Dr. Bateman, who, besides his well known intimate acquaintance with medicine, appears to have devoted a considerable degree of attention to cutaneous disorganization in particular, possessed the great advantage of enjoying enjoying the confidence of Dr. Willan, witnessing his practice, and discussing his peculiar opinions and arrangement, has, with a zeal and promptitude which must ensure success, got out the volume before us.

In the preface he cautions us not to consider it as the completion of Dr. Willan's treatise. "Its sole purpose," he observes, "is to present an abstract of the classification proposed by that respected author, together with a concise view of all the genera and species which he intended that it should comprehend. The materials for the description of the first four orders have been obtained principally from Dr. Willan's publication, of which the first part of this synopsis may be regarded as an abridgment. Some additional facts, however, have been supplied from subsequent observation. The remainder of the matter has been derived partly from personal experience and research; but principally from a constant intercourse with Dr. Willan upon the subject of these diseases, during a period of ten years, while his colleague at the Public Dispensary, and from his own communications in his last illness."

Before quoting the definitions, it is right to state that a colored engraving, which for the subject is certainly beautiful, exhibits the eight forms of cutaneous eruptions, and also illustrates some of the genera and species in a very happy manner.

DEFINITIONS.

"1. Papula (*Pimple*), a very small and acuminated elevation of the cuticle, with an inflamed base, very seldom containing a fluid, or suppurating, and commonly terminating in scurf.*

"2. Squama (*Scale*), a lamina of morbid cuticle, hard, thickened, whitish, and opaque. Scales, when they increase into irregular layers, are denominated crusts.

"3. Exanthema (*Rash*), superficial red patches, variously figured, and diffused irregularly over the body, leaving interstices of a natural color, and terminating in cuticular exfoliations.

"4. Bulla (*Bleb*), a large portion of the cuticle detached from the skin by the interposition of a transparent watery fluid.

"* The term, Papula, has been used in various acceptations by the older writers, but the nosologists have nearly agreed in restricting it to the sense here adopted. Sauvages defines it, 'Phyma parvulum, desquamari solitum.' (Nosol. Meth. class i. Synops. ord. ii. 6. See also Linnæi Gen. Morbor. class xi. ord. 4.)—In this sense also Celsus seems to have understood the term, although he uses it generally; for when he calls it a disease in which 'the skin is made *rough* and *red* by very minute pustules,' he means obviously dry papulæ; as by the word pustula he understands every *elevation* of the skin, including even wheals. (De Med. lib. v. cap. 28, § 15 and 18.)"

"5. Pus-

"5. Pustula *(Pustule)*, an elevation of the cuticle, with an inflamed base, containing *pus*.

"Four varieties of pustules are denominated in this arrangement as follows:

"*a. Phlyzacium*, a pustule commonly of a large size, raised on a hard circular base, of a vivid red color, and succeeded by a thick, hard, dark-colored scab.*

"*b. Psydracium*, a small pustule, often irregularly circumscribed, producing but a slight elevation of the cuticle, and terminating in a laminated scab.† Many of the psydracia usually appear together, and become confluent; and, after the discharge of pus, they pour out a thin watery humor, which frequently forms an irregular incrustation.

"*c. Achor*, and

"*d. Favus.* These two pustules are considered by the majority of writers from the Greeks downwards, as varieties of the same genus, differing chiefly in magnitude.‡ The *achor* may be defined a small acuminated pustule, containing a straw-colored matter, which has the appearance and nearly the consistence of strained honey, and succeeded by a thin brown or yellowish scab. The *favus*, or κηριον, is larger than the *achor*, flatter, and not acuminated, and contains a more viscid matter; its base, which is often irregular, is slightly inflamed; and it is succeeded by a yellow, semi-transparent, and sometimes cellular scab, like a honey-comb, whence it has obtained its name.

"6. Vesicula *(Vesicle)*, a small orbicular elevation of the cuticle, containing lymph, which is sometimes clear and colorless, but often opaque, and whitish or pearl-colored. It is succeeded either by scurf, or by a laminated scab.

---

"* The derivation of this term, 'απο τε φλυω, φλυζω, sive φλυσσω, quod *fervere* significat, et *ebullire*,' (Gorræi Def. Med.) would render it sufficiently appropriate to elevated and inflamed pustules, if we had not possessed also the interpretation left by Celsus: 'φλυζακιον autem paulo durior pustula est, subalbida, acuta; ex qua quod exprimitur, humidum est. Ex pustulis vero nonnunquam etiam ulcuscula fiunt, aut aridiora, aut humidiora: et modo tantum cum prurigine, modo etiam cum inflammatione aut dolore; exitque aut pus, aut sanies, aut utrumque. Maximeque id evenit in ætate puerili; raro in medio corpore; sæpe in eminentibus partibus.' (De Medicina, lib. v. cap. 28, § 15.)"

"† As the *Phlyzacia* were denominated from the heat of the eruption, so the *Psydracia* received their appellation from the opposite quality, 'quasi ψυχρα υδρακια, id est, *frigidæ* seu frigefactæ *guttulæ*,' says Gorræus.—The psydracia are enumerated among the eruptions peculiar to the head by Alexander and Paul, and some other Greek writers; but Galen and others mention them as common to other parts of the body. (See Alex. Trall. Op. lib. i. cap. 5. Paul. Ægin. lib. iii. cap. 1. Actuarius, lib. vi. cap. 2.)"

"‡ See Aëtius, tetrab. ii. serm. ii. cap. 68.—Alex. Trall. lib. i. cap. 8 and 9.—Paul. Ægin. de Re Med. lib. iii. cap. 3.—Oribas de Loc. Affect. lib. iv. cap. 12."

"7. Tu-

"7. TUBERCULUM (*Tubercle*), a small, hard, superficial tumor, circumscribed, and permanent, or suppurating partially.

"8. MACULA (*Spot*), a permanent discoloration of some portion of the skin, often with a change of its texture.

"The following terms are used in their ordinary acceptation, viz.

"9. *Wheal*, a rounded or longitudinal elevation of the cuticle, with a white summit, but not permanent, nor containing a fluid, nor tending to suppuration.

"10. FURFUR (*Scurf*), small exfoliations of the cuticle, which occur after slight inflammatioa of the skin, a new cuticle being formed underneath during the exfoliation.

"11. SCAB, a hard substance covering superficial ulcerations, and formed by a concretion of the fluid discharged from them.

"12. STIGMA, a minute red speck in the skin, without any elevation of the cuticle. When stigmata coalesce, and assume a dark-red or livid color, they are termed *Petechiæ*."

As many of our readers may not be acquainted with Dr. Willan's arrangement, we shall lay it before them, and then subjoin a specimen of Dr. Bateman's valuable performance, which ought to be in every one's hands.

"The diseases of the skin were arranged by Dr. Willan in eight orders, according to their external forms above defined, as in the following table.

*Order* I.—PAPULÆ.

| | |
|---|---|
| Strophulus. | Prurigo. |
| Lichen. | |

II.—SQUAMÆ.

| | |
|---|---|
| Lepra. | Pityriasis. |
| Psoriasis. | Ichthyosis |

III.—EXANTHEMATA.

| | |
|---|---|
| Rubeola. | Roseola. |
| Scarlatina. | Purpura. |
| Urticaria. | Erythema. |

IV.—BULLÆ.

| | |
|---|---|
| Erysipelas. | Pompholyx. |
| Pemphigus. | |

V.—PUSTULÆ.

| | |
|---|---|
| Impetigo. | Variola. |
| Porrigo. | Scabies. |
| Ecthyma. | |

VI.—VESICULÆ.

| | |
|---|---|
| Varicella. | Miliaria. |
| Vaccinia. | Eczema. |
| Herpes. | Aphtha. |
| Rupia. | |

Order

VII.—Tubercula.

Phyma.
Verruca.
Molluscum.
Vitiligo.
Acne.
Sycosis.
Lupus.
Elephantiasis.
Frambœsia.

VIII.—Maculæ.

Ephelis.

Nævus, Spilus, &c.

Where every part of a work is excellent, we are spared the necessity of criticism, and can have no hesitation about selecting specimens, for there is no occasion for choice. We cannot abridge, for Dr. Bateman has condensed his information as much as possible: it only remains for us, then, to recommend the work in the most unqualified manner. At the same time we must not be supposed to insinuate that the work is perfect, for we have no doubt that the learned author will very shortly acquire still more extensive practical knowledge upon the subject; but we consider it the only book extant that contains a comprehensive yet explicit account and scientific arrangement of the diseases of the skin; keeping always in mind that Dr. Willan's great work was only half completed.

We think Dr. Bateman might have said more upon Lupus, for in proportion as the disease is rare, and of difficult cure, he should have exerted himself to collect more particulars than he has accomplished. His alleged reason appears to us merely a *ruse* to avoid delay in getting out his book: he hastily observes, "Of this disease (Lupus) I shall not treat at any length, for I can mention no medicine which has been of any essential service in the cure of it; and it requires the constant assistance of the surgeon, in consequence of the spreading ulcerations in which the original tubercles terminate." We have seen a very severe case of this formidable disease, which had extended over nearly half the body, and was gradually advancing, completely checked, and finally destroyed, by the application of caustic. We have no doubt Dr. Bateman will have more to say upon this disease on a future occasion, and that he will not slur over the diseases resembling syphilitic appearances, although not always connected with venereal poison, as his laudable haste in furnishing the public with the present volume has probably induced him to do on this occasion. *Non omnia possumus omnes*, and Dr. B. has atchieved more in a given time than his warmest friends could have expected. He will therefore, after the manner in which we have stated the impression his book made upon us, no doubt pardon these hints.

Some readers will not purchase without a sample, and, as we before observed, the work will not admit of any curtailment, we shall copy the author's account of Lepra, the first genus of the second order SQUAMÆ.

" The term Lepra is here appropriated solely to the leprosy of the Greeks, as described by the more accurate of those writers. It is characterised by ' scaly patches, of different sizes, but having always nearly a circular form.*'

" 1. Lepra *vulgaris*, the ordinary species of the disease in this country, commences with small, round, reddish, and shining elevations of the skin, at first smooth, but within a day or two exhibiting thin white scales on their tops. These gradually, sometimes rapidly, dilate to the size of half-a-crown, still retaining their oval or circular form, and are covered with shining scales, and encircled by a dry, red, and slightly elevated border. In some cases these scales accumulate so as to form thick prominent crusts. If the scales or crusts are removed, the skin appears red and shining, being very smooth, and free from the cuticular lines in the beginning, but marked, in the advanced stages, with long deep lines and reticulations, not always coinciding with those of the adjoining surface.

" The lepra most commonly commences on the extremities, where the bones lie nearest to the surface, especially below the elbow and the knee, and usually on both arms, or both legs, at the same time. From these points it gradually extends, by the formation of new and distinct patches, along the arms or thighs, to the breast and shoulders,

" * The confusion which has every where prevailed in the use of the terms *lepra* and *leprosy*, seems to have originated principally with the translators of the Arabian writers after the revival of learning. The Greeks agreed in appropriating the appellation of λεπρα to a *scaly* eruption (as its etymology dictated): most of them deemed it the highest degree of *scaliness*, exceeding in this respect the Lichenes, Psora, and Alphos; and those who were most minute in their description, stated that ' it affects the skin deeply, in *circular* patches, at the same time throwing off scales like those of large fishes.' (See Paul. Ægin. de Re Med. lib. iv. cap. 2; and Actuarius, de Meth. Med. lib. ii. cap. 11: also Aëtius, tetrab. iv. serm. i. cap. 134; and Galen. Isagoge.) This was sufficiently clear; but those who translated the works of the Arabians into Latin, fell into the extraordinary mistake of applying the Greek term to a *tubercular* disease, which had been actually described by the Greeks under the appellation of Elephantiasis; and they applied the barbarous term *Morphœa*, together with Scabies and Impetigo, to the scaly diseases of the Greeks above enumerated. Whence their followers, who detected the error, spoke of the Lepra Arabum as well as the Lepra Græcorum; while the less accurate confounded every foul cutaneous disease under the term leprosy. The Arabians themselves do not employ the word Lepra, but have described these different diseases under appropriate appellations."

and and to the loins and sides of the abdomen. In several cases I have observed the eruption most copious and most permanent round the whole lower belly. The hands also become affected, and in many cases the hairy scalp; but the face is seldom the seat of large patches, although some scaliness occasionally appears about the outer angles of the eyes, and on the forehead and temples, extending from the roots of the hair. In the more severe cases, the nails of the fingers and toes are often much thickened, and become opaque and of a dirty yellowish hue, and are incurvated at the extremities: their surface is also irregular, from deep longitudinal furrows, or elevated ridges.

"When the eruption of lepra is moderate in degree and extent, it is not attended with any uneasy sensations, except a slight degree of itching when the patient is heated by exercise, or becomes warm in bed; and a little occasional tingling in certain states of the atmosphere.* When it is generally diffused, however, and there is a considerable degree of inflammation in the skin, it is accompanied with extreme soreness, pain, and stiffness, which I have sometimes seen so great as to render the motions of the joints impracticable, and to confine the patient to bed. Yet even under these circumstances there is no constitutional disturbance; and if no medicine be employed, the disease of the skin may continue for months, or even years, without any material derangement of the system.

"It is not easy to point out the causes of this disease, which appear, indeed, to be very various; for it is one of the most common affections of the skin, at least in this metropolis, and occurs at all periods, and under every circumstance of life.† It is certainly not communicable by contagion, nor does it appear to originate from confinement to certain kinds of diet, such as fish, dried or salted meats, &c. since it is not endemic in districts where these are habitually used, and occurs frequently where they are almost unknown. But, like some other cutaneous affections of a more transient character, it is certainly produced occasionally by the influence of particular articles of food and drink, which operate through the idiosyncrasy of individuals. I have met with one gentleman in whom spices or alcohol speedily produce it. The original attack in him occurred after eating

"* Hippocrates remarks that some *Lepræ* itch before rain: lib. Περι Χυμων."

"† It is difficult, therefore, to account for the opinion expressed by the late Dr. Heberden, respecting the extreme rarity of lepra in this country. 'De vero scorbuto et lepra, nihil habeo quod dicam, cum alter rarissimus est in urbibus, altera in Anglia pene ignota; unde factum est ut hos morbos nunquam curaverim.' (Comment. cap. 23.) And still more difficult to explain the statement of Dr. Cullen, whose definition of lepra will include both the dry and humid tetters (Psoriasis and Impetigo) with the proper scaly lepra; but who nevertheless affirms that he had never seen the disease. Nosol. Meth. class iii. gen. 88, *note*."

some hot soup, containing spice, the first spoonful of which excited a violent tingling over the whole head, which was followed by the leprous eruption, which soon extended to the limbs. In another case, in a young gentleman of nineteen, the disease commenced after taking copious draughts of cream; and vinegar, oatmeal, and other species of food, to which it has been ascribed, have probably given rise to it occasionally: but these are all anomalies, and are only referable to peculiar idiosyncracy.* In some cases it has commenced after violent and continued exercise, by which the body had been much heated and fatigued.

"Dr. Willan has imputed the origin of lepra to cold and moisture, and to certain dry sordes on the skin. It has seldom occurred to me, however, to witness the disease in bakers, laboratory men, and others who work among dry powdery substances; while I have observed a considerable number of cases in young ladies, and in persons of both sexes in respectable ranks of life, by whom every attention to cleanliness was scrupulously paid. Where cold and moisture have excited the eruption of lepra, the predisposition to it must have been peculiarly great. On the whole, the causes of this disease are involved in much obscurity. There is obviously an hereditary predisposition to it in some individuals.

"2. Lepra *alphoides*†. This is a less severe form of the disease than

"* Some poisonous substances taken into the stomach have produced an eruption of lepra. The poison of copper is stated to have speedily excited it in several persons at the same time, in one of whom it continued for a month, but disappeared in the others in about ten days. See Med. Facts and Obs. vol. iii. p. 61."

"† The Greeks have described the *Alphos* as a milder disease, being more superficial, and less rough, than the lepra: (see Galen, de Sympt. Caus. lib. iii.—Aët. tetrab. iv. serm. i. cap. 134.) and the description of it given by Celsus accords with the appearances of the L. alphoides above stated. 'Αλφος vocatur, ubi color albus est, fere subasper, et non continuus, ut quædam quasi guttæ dispersæ videantur. Interdum etiam latius, et cum quibusdam intermissionibus, serpit.' (de Medicina, lib. v. cap. 28.) Celsus no where employs the term lepra.

"This scaly *Alphos*, which was deemed by Hippocrates a blemish, rather than a disease (Περι Παθων, sect. 15), was distinguished from another *white* affection of the skin, the *Leuce*, which was not scaly, but consisted of smooth shining patches, on which the hairs turned white and silky, and the skin itself, and even the muscular flesh underneath, lost its sensibility. The *Leuce* was a disease of an incurable nature. (Hipp. Προῤῥητικ. lib. ii.) Celsus, although pointing out this distinction, includes the Leuce and the Alphos under the same generic title, Vitiligo. (loc. cit.)

"It may be remarked that the Arabians distinguished these two affections by different generic appellations, calling the Alphos *Albohak*, and than the preceding. It differs chiefly in the small size of the patches, which seldom extend beyond the diameter of a few lines, or become confluent,—in the minuteness and greater whiteness of the scales,—and in its limitation to the extremities. This variety of lepra is most common in children. It is tedious and difficult of cure, like the former, and requires similar treatment.

"It would be superfluous to enumerate the catalogue of useless medicines which have been recommended from ancient times for the cure of lepra: I shall, therefore, confine my attention to those, of the beneficial agency of which I can speak from experience. It is necessary to premise, however, that there is no one remedy, nor any invariable plan of treatment, which will succeed in lepra, under all the circumstances of its appearance in different instances; and that great errors are committed by prescribing for the name of the disease. The circumstances to which I allude more particularly, are the different degrees of cutaneous excitement, or inflammatory action, which accompany the disease in different habits; and which, if carefully attended to, afford an important guide to the most successful application of remedies.

"In the less irritable conditions of the leprous eruption, such as the L. alphoides usually exhibits, as well as a few cases of the L. vulgaris, a gently stimulant mode of treatment, at least externally, is requisite; though in all cases of lepra the diet should be light and moderate, and heating liquors should be avoided, especially malt liquors and spirits; for every indulgence in these points will be felt in the aggravation of the symptoms. A frequent use of the warm bath, with which a moderate degree of friction may be combined, contributes to remove the scales, and to soften the skin; or, if the eruption be confined to the extremities, local ablution and friction may be sufficient. These cases are benefited by the use of the sulphur waters of Harrowgate, Leamington, Crofton, and other well-known springs, both internally and externally, and by the warm sea-water bath. In fact, these gently-stimulant ablutions are often sufficient, if persevered in during several weeks, to remove the modifications of lepra of which I am now speaking.

"But if the scales adhere tenaciously, or are accumulated into thick crusts (see Def. 2), then some more active lotion must be con-

---

and the Leuce *Albaras*, with the epithet *white*. Their translators have called the former *Morphœa*, and included the Leuce and Elephantiasis under the appellation of *Lepra*. By retaining these distinctions in recollection, the accounts of the older writers may be read, while the confusion arising from their misapplication of names may be avoided.

"It appears probable, that the *Leuce* was the leprosy of the Jews, described in Leviticus, chap. xiii. See Greg. Horstii Obs. Med. lib. vii. p. 330.—Leon. Fuchsii Paradox. lib. ii. cap. 16.—Th. Campanellæ Ord. Medic. lib. vi. cap. 23.—Hensler, vom Abendländischen Aussatz, p. 341."

joined joined with the warm ablution, or with the application of steam, in order to clear the surface. Lotions of diluted alcohol, of sulphurated potass, or the decoction of dulcamara, will aid the exfoliation; and the thick crusts may be softened and loosened by lotions containing a portion of the liquor potassæ, or of the muriatic acid. When these are removed, the cuticle may be restored gradually to its healthy condition, by the unguentum picis, or the unguentum hydrargyri nitratis diluted with saturnine cerate, or simple ointment; or lotions containing a small proportion of the oxymuriate of mercury may be substituted. The ointments should be applied at night, and washed off in the morning with warm water, or a slight saponaceous lotion.

"The same cases will be accelerated in their progress towards a cure, by the use of those internal remedies which tend to support the strength, and to stimulate the cutaneous vessels. For this purpose the arsenical solution,* recommended by Dr. Fowler, is often extremely beneficial, in doses of four or five drops, which may be slowly increased to eight, and persevered in for a month or more.† Pitch, administered in the form of pills, is productive of a similar good effect, where the cutaneous circulation is very inert; but both these medicines are liable to aggravate the eruption, where it is connected with much irritability of the skin. The solution of oxymuriate of mercury has appeared to have some efficacy in these inert states; and in thin and delicate girls, of relaxed habit, affected with the lepra alphoides, the vinum ferri, or the tartrite before-mentioned, has been taken with much advantage.‡

"One of the most effectual remedies for lepra, however, under all its varieties, is the decoction of the leaves and twigs of the solanum dulcamara, which was introduced to the notice of British practitioners by Dr. Crichton.§ This medicine is at first administered in doses of

"* Preparations of this mineral have a direct tendency to stimulate the cutaneous circulation, and to inflame the skin; and are, therefore, altogether inadmissible in the irritative forms of lepra."

"† This active medicine being now not only sanctioned by the profession in general, but by the Pharmacopœia of the College, it will be enough to state, that, in these smaller doses, which experience has proved to be sufficient, it may be taken without any inconvenience. Another preparation, introduced by the late Dr. de Valangin, is kept at Apothecary's Hall, under the name of solutio solventis mineralis, and is equally efficacious in smaller doses."

"‡ If in any case the tinct. lyttæ prove useful in lepra, it would probably be in these more inert instances. But it is to be observed that Dr. Mead, who originally recommended this medicine, was speaking, not of the scaly Lepra, but of the Leuce, or of the Elephantiasis. See his Medicina Sacra, cap. ii."

"§ See his communication to Dr. Willan. (Treatise on Cutan. Diseases, p. 145.) His formula has been adopted by the College in the late edition of the Pharmacopœia."

two or three ounces thrice every day, which are gradually augmented, until a pint is at length consumed daily. When there is a degree of torpor in the superficial vessels, the same decoction, made with a larger proportion of the shrub, is advantageously employed as a lotion; but if there is much inflammatory disposition, this and every other external stimulus must be prohibited.

"Where this irritable state of the disease exists, indeed, (and it is the most frequent,) nothing more stimulating than tepid water, or thin gruel, can be used for the purposes of ablution; and the arseniates, pitch, &c. above mentioned, must be excluded. The disease, under this condition, will be certainly aggravated by sea-bathing, by the external use of the strong sulphureous waters, or of any irritant, as I have frequently observed; but it will be alleviated by the internal employment of sulphur, with soda or nitre, or the hydrarg. sulphuratus niger with an antimonial, especially when conjoined with the decoction of dulcamara. The caustic potass, or liquor potassæ of the L. Pharmacopœia, in the dose of twenty or thirty drops, alone, or in combination with the precipitated sulphur, is likewise beneficial; and the tinctura veratri, given in such doses as not to disorder the bowels, has occasionally removed this state of the disease.

"When the skin is highly inflamed, thickened, and stiff, of a vivid red color, intermixed with a yellowish hue, (where the cuticle is separating in large flakes,) the heat, pain, and itching are often extremely troublesome, and the motion of the limbs is almost impracticable. The most effectual relief is obtained, in these cases, by gently besmearing the parts with cream, or a little fresh and well washed lard, or butter.

"3. Lepra *nigricans* is a more rare variety of the disease, differing externally from the L. vulgaris chiefly in the dark and livid hue of its patches, which is most obvious in the margin, but even appears through the thin scales in the area of each patch.* The scales are more easily detached in this form of lepra, and the surface remains longer tender, and is often excoriated, discharging bloody serum, till a new incrustation is formed.

"This variety of lepra occurs in persons whose occupations expose them to the vicissitudes of the weather, and to a precarious diet, with fatigue, and watching. It is cured by nutritive food, with moderate exercise, followed by the use of the bark, mineral acids, and sea-bathing."

---

* The *Melas* of the ancients was deemed a superficial affection, resembling the Alphos, except in its color. 'Μελας colore ab hoc differt, quia niger est, et umbræ similis: cætera eadem sunt.' (Celsus, loc. cit.) Possibly it included the Pityriasis versicolor."

MEDICAL

